# Ovarian Suppression: Early Menopause and Late Effects

**DOI:** 10.1007/s11864-024-01190-8

**Published:** 2024-03-13

**Authors:** Chiara Molinelli, Flavia Jacobs, Guilherme Nader-Marta, Roberto Borea, Graziana Scavone, Silvia Ottonello, Piero Fregatti, Cynthia Villarreal-Garza, Jyoti Bajpai, Hee Jeong Kim, Silvia Puglisi, Evandro de Azambuja, Matteo Lambertini

**Affiliations:** 1 Department of Internal Medicine and Medical Specialties (DiMI), School of Medicine, University of Genova, Genoa, Italy; 2 Department of Medical Oncology, U.O. Clinical Di Oncologia Medica, IRCCS Ospedale Policlinico San Martino, Largo Rosanna Benzi 10, 16132 Genoa, Italy; 3 Humanitas Clinical and Research Center – IRCCS, Humanitas Cancer Center, Via Manzoni 56, 20089 Rozzano, Milan Italy; 4 Academic Trials Promoting Team, Institut Jules Bordet and l’Université Libre de Bruxelles (U.L.B), 90, Rue Meylemeersch, 1070 Anderlecht, Brussels Belgium; 5 Department of Surgery, U.O. Senologia Chirurgica, IRCCS Ospedale Policlinico San Martino, Genoa, Italy; 6 Department of Surgical Sciences and Integrated Diagnostic (DISC), School of Medicine, University of Genoa, 16132 Genoa, Italy; 7 Breast Cancer Center, Hospital Zambrano Hellion - TecSalud, Tecnologico de Monterrey, Monterrey, Mexico; 8 Tata Memorial Centre, Homi Bhabha National Institute (HBNI), Ernest Borges Rd, Parel East, Parel, Mumbai, Maharashtra 400012 India; 9 Division of Breast Surgery, Department of Surgery, Asan Medical Center, University of Ulsan College of Medicine, 88, Olympic-Ro 43-Gil, Songpa-Gu, Seoul, South Korea; 10 Medical Oncology Unit 1, IRCCS Ospedale Policlinico San Martino, Largo Rosanna Benzi 10, 16132 Genoa, Italy

**Keywords:** Breast cancer, Ovarian suppression, Endocrine treatment, Young patients

## Abstract

Around 90% of breast tumours are diagnosed in the early stage, with approximately 70% being hormone receptor-positive. The cornerstone of adjuvant therapy for early-stage hormone receptor-positive breast cancer is endocrine therapy, tailored according to disease stage, biological characteristics of the tumour, patient’s comorbidities, preferences and age. In premenopausal patients with hormone receptor-positive breast cancer, ovarian function suppression is a key component of the adjuvant endocrine treatment in combination with an aromatase inhibitor or tamoxifen. Moreover, it can be used during chemotherapy as a standard strategy for ovarian function preservation in all breast cancer subtypes. In the metastatic setting, ovarian function suppression should be used in all premenopausal patients with hormone receptor-positive breast cancer to achieve a post-menopausal status. Despite its efficacy, ovarian function suppression may lead to several side effects that can have a major negative impact on patients’ quality of life if not properly managed (e.g. hot flashes, depression, cognitive impairment, osteoporosis, sexual dysfunction, weight gain). A deep knowledge of the side effects of ovarian function suppression is necessary for clinicians. A correct counselling in this regard and proactive management should be considered a fundamental part of survivorship care to improve treatment adherence and patients’ quality of life.

## Introduction

Approximately one out of five new diagnoses of breast cancer (18%) occurs in women younger than 50 years old [1]. Young age seems to be associated with poorer survival outcomes in women affected by hormone receptor-positive breast cancer, which represents the most common subtype also in this patient population [2]. As compared to post-menopausal patients, breast cancers arising in premenopausal patients seem to harbour more aggressive features, namely higher tumour stage and more frequent luminal B-like biology within the subgroup of hormone receptor-positive tumours [3]. Despite advances in treatment modalities, more effective endocrine therapy approaches and improved understanding of tumour biology [4], many premenopausal women with hormone receptor-positive early breast cancer are still being treated with chemotherapy. Premature ovarian insufficiency (POI) is a potentially irreversible toxicity of chemotherapy in premenopausal patients, requiring appropriate counselling since diagnosis [5]. In premenopausal women, ovarian function suppression (OFS) may have a double role. On one side, it can be used during chemotherapy as a standard strategy for ovarian function preservation in all patients, regardless of breast cancer subtype [6]. On the other side, OFS is a key component of adjuvant endocrine treatment in combination with an aromatase inhibitor (AI) or tamoxifen in patients with hormone receptor-positive breast cancer [7], except for those affected by cancers at low risk of recurrence. Moreover, in the metastatic setting, OFS should be used in all patients with hormone receptor-positive breast cancer in order to achieve a post-menopausal status [8]. Ovarian function can be temporarily suppressed by using a gonadotropin-releasing hormone agonist (GnRHa) or can be permanently blocked through bilateral oophorectomy. The administration of GnRHa (e.g. goserelin, leuprolelin, triptorelin) is not invasive and it is reversible. On the contrary, oophorectomy requires surgery and leads to an irreversible OFS. Despite its key role, OFS is associated with many different and relevant side effects that can have a major negative impact on patients’ quality of life if not properly managed and may lead to premature treatment interruption. The aim of this review is to summarize the main role and efficacy data of OFS in early breast cancer as well as its side effects with a special focus on their possible management.

## Indications to OFS in Early Breast Cancer

### OFS as a Strategy for Ovarian Function Preservation During Chemotherapy

The efficacy of GnRHa administration during chemotherapy in order to preserve ovarian function in breast cancer patients has been evaluated in several randomized trials [9–23] (Table 1). Most studies enrolled a small number of patients, usually less than 100. The definition of chemotherapy-induced POI was mostly based on the absence of menstrual cycles after chemotherapy. However, in some studies, the combination of amenorrhea and post-menopausal hormonal levels was required for POI diagnosis. The evaluation was performed from 6 months up to more than 5 years after the end of treatment. Most of the patients received anthracycline- and cyclophosphamide-based chemotherapy regimens. Of note, in most of the studies, median age was around 40 years and pregnancy desire was not an inclusion criteria nor was collected for the majority of the patients. The median follow-up was relatively short in most of the trials to assess long-term endpoints like post-treatment pregnancies [24]. In the POEMS study, 218 premenopausal women affected by hormone receptor-negative early breast cancer were randomized to receive chemotherapy plus goserelin or chemotherapy alone. The risk of developing ovarian failure was reduced by 70% at 2 years after treatment (OR 0.30; 95% CI 0.09–0.97; *p* = 0.04) and more patients treated with the addition of GnRHa had a post-treatment pregnancy (23.1% vs. 12.2%, adjusted OR 2.34, 95% CI 1.07–5.11, *p* = 0.03) [19]. Consistent results were seen in the PROMISE-GIM6 study that included mostly (80%) patients with hormone receptor-positive breast cancer. In this trial, 281 premenopausal patients affected by early breast cancer who were candidates for (neo)adjuvant chemotherapy were randomly assigned to receive chemotherapy plus GnRHa triptorelin or chemotherapy alone. The chemotherapy-induced POI rate was significantly lower in patients treated with chemotherapy plus GnRHa than in those treated without GnRHa (8.9% vs. 25.9%; OR 0.28, 95% CI 0.14–0.59) [25]. At a median follow-up of 12.4 years, the cumulative incidence of pregnancy was 6.5% in the combination arm and 3.2% in patients treated with chemotherapy alone (HR 2.14, 95% CI 0.66–6.92). The authors also performed an exploratory descriptive analysis in *BRCA*-mutated patients. Among patients harbouring *BRCA* pathogenic variants, the incidence of POI was 0% in those treated with GnRHa and 33% in those treated without GnRHa. Despite the small numbers, these findings are consistent with the main trial results showing a benefit of GnRHa use during chemotherapy also among *BRCA* carriers [26]. A similar reduction in terms of POI rates (10.3% in the GnRHa group and 44.5% in the control group) was found by Zong et al. in a population of 301 patients [23]. Globally, all the studies conducted except for four [12, 14, 15, 22], demonstrated that the use of GnRHa has a protective role on ovarian function when administered during chemotherapy. Several meta-analyses have been conducted [6, 27–35]; all of them, except for one [30], showed a reduced risk of chemotherapy-induced POI in patients receiving GnRHa with chemotherapy. To date, the addition of GnRHa to chemotherapy is recommended by international guidelines as a standard strategy for ovarian function preservation in premenopausal patients affected by breast cancer [5, 36–38]. Goserelin and leuprorelin seem to be equally effective in terms of ovarian protection in young patients affected by breast cancer administered with chemotherapy [39]. No data are available regarding the comparison head-to-head between triptorelin and other GnRHa agents. Importantly, the use of GnRHa should not be considered per se a strategy for fertility preservation and should not replace cryopreservation procedures in patients interested in preserving fertility before starting chemotherapy [38].

**Table 1:** Randomized clinical trials evaluating temporary ovarian suppression with GnRHa during chemotherapy as a strategy for ovarian function preservation in premenopausal women with breast cancer. Abbreviations: *CT* chemotherapy, *DFS* disease-free survival, *E2* estradiol, *FSH* follicle-stimulating hormone, *GnRHa* gonadotropin-releasing hormone agonist, *POI* premature ovarian insufficiency, *PM* post-menopausal, *OS* overall survival

Authors	POI definition	Timing POI evaluation (months)	Treatment regimen	No. patients	Median age (years) (GnRHa vs. control)	POI rate (GnRHa vs. control)	Pregnancies
Li et al. [9]	Amenorrhea	12	CT + / − goserelin	63	40 vs. 39	32.1% vs. 53.1% (*p* = 0.027)	NA
Badawy et al. [11]	Amenorrhea and no resumption of ovulation	8	CT + / − goserelin	78	30 vs. 29.2	11.4% vs. 66.6% (*p* < 0.001)	NA
Sverrisdottir et al. [10]	Amenorrhea	Up to 36	CT + / − goserelin (+ / − tamoxifen) (+ − /tamoxifen)	94	45 vs. 45–46	64% (93%) vs. 90% (87%) (*p* = 0.006)	NA
Gerber et al. [12]	Amenorrhea	6	CT + / − goserelin	60	35 vs. 38.5	30% vs. 43.3% (*p* = 0.142)	1 vs. 1
Sun et al. [13]	Amenorrhea	12	CT + / − goserelin	21	38 vs. 37	27.3% vs. 50.0% (*p* = 0.039)	NA
Lambertini et al. [20]	Amenorrhea and PM levels of FSH and E2	12	CT + / − triptorelin	281	39 vs. 39 39	8.9% vs. 25.9% (*p* < 0.001)	8 vs. 3 (*p* = 0.20)
Munster et al. [14]	Amenorrhea	24	CT + / − triptorelin	49	39 vs. 38	15% vs. 14% (*p* = 0.32)	0 vs. 2
Elgindy et al. [15]	Amenorrhea	12	CT + / − triptorelin (± GnRHa antagonist)	100	33 vs. 32	20%/16% vs. 20%/20% (*p* = 1.00/*p* = 0.71)	2 vs. 1
Song et al. [16]	Amenorrhea and PM levels of FSH and E2	12	CT + / − leuprolide acetate	183	40.3 vs. 42.1	16.9% vs. 28.7% (*p* < 0.01)	NA
Jiang et al. [17]	Amenorrhea	NA	CT + / − triptorelin	32	NA	10.0% vs. 45.5% (*p* = 0.05)	NA
Karimi-Zarchi et al. [18]	Amenorrhea	6	CT + / − triptorelin	42	37	9.5% vs. 66.7% (*p* < 0.001)	NA
Moore et al. [19]	Amenorrhea and PM levels of FSH	24	CT + / − goserelin	218	37.6 vs. 38.7	8% vs. 22% (*p* = 0.04)	22 vs. 12 (*p* = 0.03)
Leonard et al. [21]	Amenorrhea and PM levels of FSH	12–24	CT + / − goserelin	221	37.9 vs. 38.8	18.5% vs. 34.8% (*p* = 0.048)	7 vs. 5
Zhang et al. [22]	Amenorrhea and PM levels of FSH and E2	36–72	CT + / − goserelin	216	37.5 vs. 39	23.1% vs. 22.8% (*p* = 0.969)	NA
Zong et al. [23]	AMH < 0.5 ng/mL	12	CT + / − goserelin or leuprolelin	301	40.6 vs. 40.2	10.3% vs. 44.5% (*p* =)	NA

### OFS as Adjuvant Endocrine Treatment in Early Breast Cancer

The cornerstone of adjuvant treatment for hormone receptor-positive early breast cancer is endocrine therapy, tailored according to the disease stage, tumour’s biological characteristics, patients’ comorbidities, preferences and age [40–42]. Premenopausal women, especially those diagnosed before age 40 years, tend to have poorer long-term outcomes [43]. Several factors contribute to this age-related disparity, including advanced disease stage at diagnosis, less favourable disease characteristics, higher rates of side effects contributing together with other factors to a suboptimal adherence to endocrine treatment [44]. Over the past two decades, several studies evaluated the efficacy of combining OFS with endocrine treatment.

The Suppression of Ovarian Function Trial (SOFT) and the Tamoxifen and Exemestane Trial (TEXT) enrolled premenopausal women with hormone receptor-positive early breast cancer [45, 46]. SOFT aimed to determine the role of adding OFS to tamoxifen or exemestane. TEXT aimed to compare the efficacy of exemestane or tamoxifen in women undergoing OFS [45, 46]. In the SOFT trial, at a median follow-up of 12 years, patients treated with tamoxifen plus OFS had better DFS (76.1% vs. 71.9%; HR 0.82; 95% CI, 0.69–0.98) and OS (89.0% vs. 86.8%; HR, 0.78; 95% CI, 0.60–1.01) when compared to those treated with tamoxifen alone. The benefit of adding OFS was observed primarily in patients treated with chemotherapy (DFS: 78.8% in patients treated with tamoxifen, 81.1% in those treated with tamoxifen plus GnRHa, 89.4% in women administered with exemestane plus GnRHa; OS: 78.8%, 81.1%, and 84.4%, respectively) [47]. A combined analysis of SOFT and TEXT was designed to investigate the efficacy of exemestane plus OFS as compared to tamoxifen plus OFS. At a median follow-up of 13 years, the exemestane plus OFS group exhibited higher DFS rates, with an absolute improvement of 4.6% (HR 0.79; 95% CI, 0.70–0.90; *p* < 0.001). Additionally, this group reported higher proportions of patients who remained free from distant recurrence, with an absolute improvement of 1.8% (HR, 0.83; 95% CI, 0.70–0.98; *p* = 0.03). No significant difference was observed in terms of OS (90.1% vs 89.1%; HR, 0.93; 95% CI, 0.78–1.11). The benefit was greater in patients at higher risk of recurrence (patients with young age, tumour size > 2 cm, G3 tumours) [48].

Several additional studies have investigated the use of tamoxifen or an AI combined with OFS in premenopausal patients (Table 2) [49–52]. In the E-3193 trial, 345 premenopausal patients with low-risk breast cancer (node-negative, hormone receptor-positive, tumour size ≤ 3 cm) were randomized to receive tamoxifen alone or tamoxifen plus OFS. At a median follow-up of 9.9 years, no significant difference was found between the two groups for DFS (5-year rate: 87.9% vs. 89.7%; *p* = 0.62) or OS (5-year rate: 95.2% vs. 97.6%; *p* = 0.67). Notably, ≥ G3 toxicity was more frequent in the combination arm (22.4% v 12.3%) [52]. On the contrary, in the ASTRRA study, the addition of 2-year OFS to tamoxifen in premenopausal patients who had received chemotherapy (and therefore considered at higher risk) and resumed ovarian function within 2 years from its completion led to an improvement in the 8-year iDFS rate (85.4% vs. 80.2%, HR 0.67, 95% CI 0.51–0.87) [53]. These data confirm that the addition of OFS to tamoxifen should be considered in the majority of premenopausal patients with hormone receptor-positive breast cancer with the exception of those considered at low risk of disease recurrence.

For the tamoxifen vs. AI question in patients receiving OFS, a meta-analysis conducted by the EBCTCG analysed data from the four trials (ABCSG-12, TEXT, SOFT, HOBOE). This analysis including data from over 7000 patients revealed that premenopausal women undergoing OFS plus an AI had a reduced risk of recurrence compared to those treated with tamoxifen (RR 0.79, 95% CI 0.69–0,90, *p* = 0.0005 with the greatest benefit observed in the first 4 years. However, no difference was found in terms of OS [54]. Among these trials, ABCSG-12 was the only negative study showing no DFS difference between an AI and tamoxifen added to OFS. However, notably, this trial included mostly premenopausal patients with low-risk breast cancer (i.e. 67% had node-negative tumours) and the endocrine treatment was administered only for 3 years.

The selection of the most suitable endocrine treatment depends on multiple factors. In the SOFT and TEXT trials, a sophysticated analysis revealed that in women with the lowest risk of recurrence (who did not receive prior chemotherapy), there was no difference in terms of survival outcomes between the different endocrine regimens [55]. Thus, tamoxifen alone still remains the standard of care in these women [56]. Among patients with the highest risk of recurrence, the combination of exemestane plus OFS showed a superior benefit as compared to tamoxifen. When deciding on adjuvant endocrine treatment, it is crucial to consider and discuss with patients the absolute risk of disease recurrence, potential benefits, and possible side effects of the different options. The Regan risk score is an online tool that incorporates age, lymph node status, and tumour grade and can assist in estimating the risk of distant recurrence [57]. This tool is helpful in identifying patients with high-risk of disease recurrence who may benefit from escalated endocrine therapy, as well as those with low-risk who can safely be treated with tamoxifen alone, thereby avoiding unnecessary toxicities and maintaining a good quality of life [56].

Both oral endocrine agents and OFS can have adverse effects, including gynecological, sexual, musculoskeletal, and psychological events. Early identification and effective management of these adverse events are crucial to prevent treatment discontinuation. Findings from the SOFT trial revealed that rates of non-adherence to OFS increased over time, with higher risk of treatment discontinuation in very young women [58]. It is worth noting that women undergoing chemotherapy-induced amenorrhea and entering menopause are at risk of reverting to premenopausal status when treated with an aromatase inhibitor alone. This risk is more pronounced in younger premenopausal women, particularly those under the age of 50 years and is influenced by other factors including the duration and type of chemotherapy received. It is recommended to regularly monitor estradiol levels to confirm menopausal status [42]. An important unanswered question is whether OFS can replace adjuvant chemotherapy in intermediate-risk, endocrine-responsive early breast cancer. The use of genomic assays, such as OncotypeDX and MammaPrint, has revolutionized adjuvant treatment decisions in BC, but their applicability in premenopausal patients, especially those under 40 years with node-positive disease, is still debated [59, 60]. This is mainly because trials testing genomic assays have had a limited representation of women under the age of 40 years and premenopausal women included in these trials predominantly received tamoxifen alone as adjuvant endocrine therapy [61]. The TAILORx and RxPONDER trials have demonstrated the benefit of adding chemotherapy to endocrine treatment for premenopausal women, except for those with low genomic risk scores [4, 62, 63]. However, it is still unclear if the benefit of chemotherapy in premenopausal women with hormone receptor-positive early breast cancer is due to the direct cytotoxic effect of chemotherapy or to the induction of chemotherapy-induced amenorrhea. The TAILORx trial showed that patients aged 46–50 years derived greater benefit from chemotherapy as compared to those aged under 40 years. This suggests that chemotherapy-induced amenorrhea probably plays a more relevant role than the direct cytotoxic effect of chemotherapy in a condition of induced and permanent OFS, which is more likely in perimenopausal women (46–50 years) than in younger women [4, 62]. The results of the pre-operative ADAPT and ADAPT-cycle trials showed that the addition of OFS to endocrine therapy resulted in a significant increase in endocrine therapy response by reducing Ki67 levels to less than 10% in premenopausal patients, regardless of Recurrence Score (0–25 and ≥ 26). Moreover, in the subgroup of women under 40 years, in which the evidence is more controversial, the addition of OFS to an AI resulted in endocrine treatment response in both low and high-risk groups. This supports the assumption that chemotherapy may be potentially omitted in favour of an optimal endocrine therapy with OFS plus endocrine treatment in some premenopausal patients with low-risk N1 early breast cancer based on clinical and genomic risk along with the response to pre-operative endocrine therapy [65-67]. Further research is needed to determine the role of OFS combined with endocrine treatment in replacing adjuvant chemotherapy in premenopausal patients. A new trial including patients with hormone receptor-positive early node negative breast cancer with intermediate genomic risk and those with 1–3 positive nodes with low-intermediate genomic risk is currently ongoing, randomizing patients to receive chemotherapy followed by GnRHa and exemestane or GnRHa and exemestane without chemotherapy (NCT05879926).

**Table 2:** Main randomized clinical trials investigating ovarian function suppression in combination with endocrine therapy (tamoxifen or an aromatase inhibitor) in premenopausal women with hormone receptor-positive early breast cancer. Abbreviations: *BC* breast cancer, *CT* chemotherapy, *ET* endocrine therapy, *HR* hazard ratio, *LHRH* luteinizing hormone-releasing hormone, *NA* not available, *N0* node negative, *N* + node positive, *OFS* ovarian function suppression, *TAM* tamoxifen, *ZOL* zoledronic acid

Trial	Number of patients	Patients population	Treatment comparison	Patients who received CT (%)	Median age (years)	Median follow-up (years)	DFS	OS
SOFT (TAM cohorts)	2045	Premenopausal patients with N0 or N +	TAM + OFS TAM x 5y	53	43	12	76.1% vs. 71.9% (HR 0.82; 95% CI, 0.69–0.98)	89% vs. 86.8% (HR 0.78; 95% CI, 0.60–1.01)
SOFT/TEXT	4690	Premenopausal patients with N0 or N +	Exemestane + OFS x 5y TAM + OFS x 5y	60	43	13	80.5% vs. 75.9% (HR 0.79; 95% CI, 0.70–0.90)	90.1% vs 89.1%, (HR 0.93; 95% CI, 0.78–1.11)
ABCSG-12	1803	Premenopausal patients with < 10 positive nodes	Anastrozole + LHRH TAM + LHRH both + ZOL x 3y	5.7	45	8.0	HR 0.77; 95% CI, 0.60–0.99; *p* = 0.042	HR 0.66; 95% CI, 0.43–1.02; *p* = 0.064
HOBOE (cohorts without ZOL)	710	Premenopausal patients with N0 or N +	Letrozole + LHRH TAM + LHRH	63	44	5.3	93.2 vs 85.4% (HR 0.72; 95% CI, 0.48–1.07)	NA
ASTRRA	1293	Premenopausal patients who resumed ovarian function after CT	TAM x 5y + OFS TAM x 5y	100	40	8.9	85.4% vs. 80.2% HR 0.67; 95% CI, 0.51–0.87)	96.5% vs. 95.3% (HR, 0.78; 95% CI, 0.49–1.25)
E-3193 (INT-0142)	345	Premenopausal patients with < 3 cm N0 not treated with CT	TAM alone TAM + OFS	0	45	9.9	87.9 vs 89.7% (HR 1.17; 95% CI, 0.64–2.12)	95.2 vs 97.6% (HR 1.19; 95% CI, 0.52–2.70)

## Side Effects

**Fig. 1:**
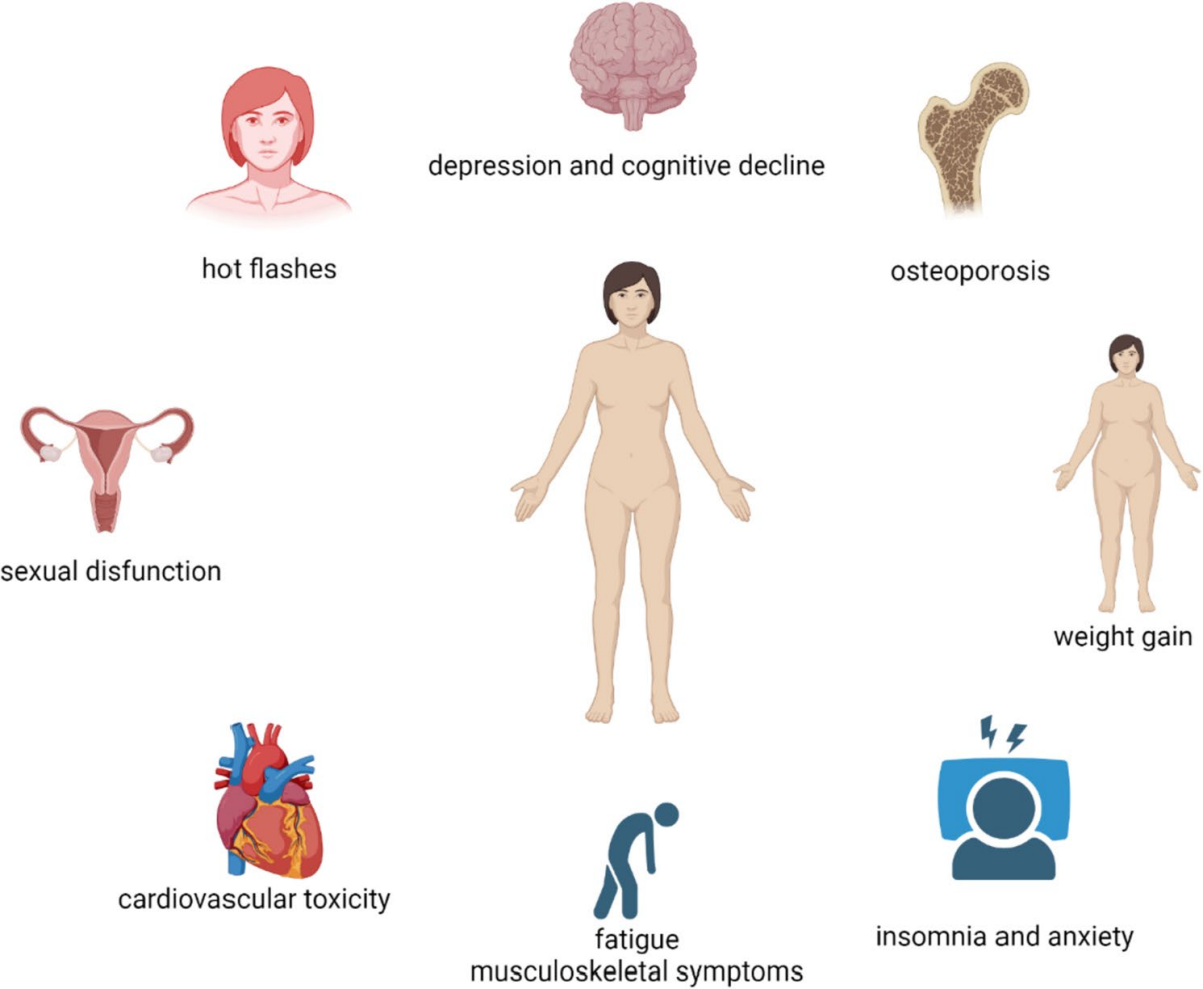
Potential side effects of ovarian function suppression. Created with BioRender.com.

Despite the relevant benefit in terms of reducing the risk of chemotherapy-induced POI and survival outcomes as adjuvant endocrine therapy, OFS is characterized by several side effects, which may lead to therapy discontinuation and poor quality of life (Fig. 1). In a combined analysis of the SOFT and TEXT trials, 31% of patients treated with tamoxifen plus OFS reported G ≥ 3 adverse events. This rate was 32.3% in those receiving exemestane plus OFS [46]. The toxicity profile of this treatment depends also on the oral endocrine agent combined with GnRHa. Arthralgia and sexual dysfunction are more frequent in patients treated with an AI, while night sweats and hot flashes are more frequent in those receiving tamoxifen [67, 68].

### Hot Flashes

Hot flashes are a group of vasomotor symptoms characterized by a sensation of warmth, flashing and perspiration in response to a hypothalamic thermoregulatory recalibration precipitated by a decline in estrogen levels [69, 70]. In this process, the core body temperature set-point is modified, triggering physiologic mechanisms to dissipate heat that cause the symptoms at lower body temperatures [69]. In the SOFT trial, the addition of OFS increased the incidence of hot flashes to 93%, compared to 80% with tamoxifen alone [45]. Although hot flashes are not life-threatening adverse events, they must be properly managed as they are associated with quality of life deterioration and reduced adherence to adjuvant treatment [71]. Evidence-based non-hormonal pharmacological strategies for managing hot flashes include the use of antidepressants and anticonvulsants. The most widely studied antidepressant agents for controlling hot flashes are selective serotonin reuptake inhibitors (SSRI) and serotonin–norepinephrine reuptake inhibitors (SNRI). Randomized studies have shown reductions of up to 60% in hot flashes with the use of venlafaxine [72]. Other agents also studied in this context include escitalopram, paroxetine and sertraline [73–75]. Importantly, some SSRI are potent CYP2D6 inhibitors and may reduce the transformation of tamoxifen to the active metabolite (endoxifen); thus, this combination should be avoided [76]. Among anticonvulsants, gabapentin and pregabalin are effective agents to attenuate hot flashes in this population [72]. Hormonal agents, although used to treat vasomotor symptoms in the general population, are contraindicated in patients with a history of breast cancer because they are associated with an increased risk of recurrence. Fezolinetant, a neurokinin 3 receptor antagonist, has been approved in the USA for menopausal symptoms; however, as of now, it has not been studied in patients with breast cancer and therefore it is not indicated [77]. Potentially useful non-pharmacological interventions in the management of hot flashes include weight control, dietary interventions and cognitive behavioral therapy [78].

### Depression/Anxiety and Sleep Disorders

Psychiatric disorders, depression and anxiety are common in cancer survivors and are associated with an increased risk of all-cause mortality [79]. A large French cohort study including data from over 4800 women with breast cancer demonstrated that nearly one third of patients experienced significant depressive symptoms during and after treatment [80]. Although the frequency of depression varies between different series, several studies suggest that OFS is associated with an increased incidence. In the SOFT trial, the incidence of any-grade depression increased from 46.6% to 51.9% with the addition of OFS to endocrine therapy [45]. Interestingly, recent evidence suggests that the incidence of major depressive symptoms may vary according to the suppression method used, with GnRHa being more commonly associated with depression than ovarian ablation [81]. The development of anxiety represents an important psychosocial issue in adult cancer survivors, with some data suggesting that it is even more frequent than depression in the long-term [82]. The impact of OFS on the incidence of anxiety is controversial, with small studies suggesting that there is no significant influence of OFS on the occurrence of anxiety [83, 84]. Depressive symptoms include emotional, cognitive, physical, and behavioral manifestations and should be actively screened in follow-up visits to avoid delays in diagnosis. The management of depression and anxiety in cancer survivors follows the same principles used in the general population, including pharmacological and non-pharmacological measures, particularly psychotherapeutic interventions, such as cognitive behavioral therapy [85]. The choice of antidepressant agent should be personalized and should consider the patients’ comorbidities, drug interactions (including with endocrine therapy), the type of depressive symptoms and the adverse effects profile of each treatment class. Pharmacological management of anxiety commonly includes the use of benzodiazepines, SSRIs, antipsychotics and neuroleptics, sometimes in combination [86]. Sleeping disorders are a related symptom whose incidence can reach up to 57% in patients under OFS [45]. Its treatment should always include a comprehensive assessment aimed at identifying potential underlying causes such as anxiety and depression, thus allowing proper management.

### Cognitive Impairment

Cognitive impairment is a known adverse effect of several oncological therapies, and may be associated with dysfunctions in multiple domains, including memory impairment and difficulty in concentrating. Both cytotoxic chemotherapies and endocrine therapies have been associated with cognitive changes [87, 88]. It is hypothesized that the cognitive effect of endocrine therapies is associated with a local reduction in estrogen levels since the expression of estrogen receptors and aromatase throughout the hypothalamus, amygdala, dorsolateral prefrontal cortex, hippocampus, areas are involved in memory, executive function and learning [89, 90]. However, studies that sought to assess the specific association between OFS and increased risk of cognitive decline showed conflicting results. A small randomized study comparing cognitive function between patients treated with tamoxifen alone or with OFS (associated with tamoxifen or exemestane) did not demonstrate a significant increase in cognitive adverse events among patients who received the combined therapy [91]. The hypothesis that chemotherapy-induced menopause would be the underlying cause of the cognitive decline associated with chemotherapy was refuted in an analysis of patient-reported outcomes of the TAILORx study, in which no significant interaction between menopausal status and cognitive impairment was demonstrated [92].

### Osteoporosis

Early menopause induced by OFS may anticipate and accelerate the development of events associated with estrogen levels drop, including bone loss [68]. In the SOFT study, the addition of OFS to endocrine monotherapy was associated with an increased risk of osteoporosis (defined as *T* score <  − 2.5) from 3.5 to 5.8% [45]. All factors associated with a decrease in endogenous estrogens, including several therapies used in the treatment of breast cancer, such as AI, surgical oophorectomy, GnRHa, chemotherapy-induced POI are associated with increased bone loss and, in some patients, increased risk of fractures [93–95]. Patients treated with OFS, particularly when associated with an AI, must undergo a complete fracture risk assessment, including the evaluation of clinical risk factors for osteoporosis (e.g. age, comorbidities, poor nutrition, low body weight, physical inactivity), as well as bone density measurement [96, 97]. Non-pharmacological measures to promote bone health should be widely encouraged, including physical activity (with weight-bearing exercise), avoiding smoking and alcohol intake and obtaining sufficient levels of calcium and vitamin D [97, 98]. Treatment of established osteoporosis in patients on OFS should be tailored according to fracture risk, patient characteristics, including comorbidities and renal function. Bisphosphonates and denosumab are standard therapies for the treatment of osteoporosis and its prevention. In premenopausal patients receiving OFS plus AI or tamoxifen with or without OFS, intravenous zoledronic acid (4 mg once every 3–6 months) should be considered the preferred choice considering the lack of evidence in this indication for denosumab [99–101]. Moreover, in both the HOBOE and the ABCSG-12 trials the use of zolendronic acid added to endocrine therapy including OFS showed to have a potential anticancer effect leading to improved outcomes [99, 102].

### Sexual Dysfunction

Sexual dysfunction is a common side effect of OFS. About 50% of breast cancer survivors report sexual dysfunction during or after the treatment. Particularly, 45% report sexual pain [103]. Sexual dysfunction includes different manifestations such as vaginal dryness, dyspareunia, decreased libido, low self-esteem, barriers on intimacy and difficulties in communicating with the partner. A comprehensive assessment and a multidisciplinary approach are needed, as well as a close collaboration with gynaecologists and psychologists [68]. Pharmacological strategies to treat sexual dysfunction include intravaginal estradiol-releasing tablets, estrogen-based vaginal creams, estradiol-releasing vaginal rings, vaginal testosterone and vaginal DHEA. These strategies reduce the effects of oestrogen deprivation, but they also seem to determine an increase in serum estradiol levels, which could be an undesirable consequence in breast cancer patients [78]. Non-hormonal strategies should be chosen in the first instance for sexual dysfunction in breast cancer survivors, particularly those with hormone receptor-positive breast cancer on adjuvant endocrine therapy. A short treatment with low-dose vaginal estrogen could be evaluated in selected patients with severe symptoms, after a careful discussion. On the contrary, different trials evaluated the efficacy of nonhormonal vaginal lubricants, concluding that they can be considered without any concerns in terms of safety [104, 105]. They represent a cost-effective strategy to reduce dyspareunia and vaginal dryness. Laser therapy represents a non-pharmacological approach with limited evidence of efficacy. One of the largest, but still retrospective, studies was conducted by Pagano et al., who treated 82 breast cancer survivors with CO2 laser for 3 cycles. They used a visual analogue scale for evaluating vaginal dryness, itching, dyspareunia and dysuria. An improvement in visual analogue scale was reported for each symptom (*p* < 0.001), regardless of age and type of endocrine treatment [106]. However, the high cost, the reduced availability and specifically the lack of randomized clinical trials represent the limits of this approach. Notably, these approaches are not FDA-approved and should not be recommended in patients with breast cancer [68]. Cognitive behavioural therapy is highly recommended in breast cancer survivors reporting sexual dysfunction. In a study conducted by Hummel et al., 169 breast cancer survivors were randomized to cognitive behavioural therapy for 24 weeks at maximum or a waiting-list control group. The experimental arm had a significant improvement in sexual functioning, sexual desire, sexual arousal, vaginal lubrication, body image and menopausal symptoms than the control group [107]. In this study, the authors performed an internet-based cognitive behavioural therapy, but similar results were found in another study using a single 4-h group intervention including sexual health rehabilitation, body awareness exercises and mindfulness-based cognitive therapy [108].

### Weight Gain

The association between obesity and poorer breast cancer prognosis is well established [109–111]. Moreover, obesity is associated with a poor quality of life and social stigma [112]. Obesity is also a risk factor for other impactful conditions, namely cardiovascular diseases, fatigue, diabetes and metabolic syndrome [113]. The mechanism of action underlying the correlation between obesity and breast cancer is partly unclear but includes increase serum estrogen levels due to hyper-adiposity, a chronic inflammation with high levels of pro-inflammatory proteins, prolonged hyperinsulinemia [114–116]. Obesity represents a risk factor for incomplete OFS in premenopausal women receiving GnRHa plus an AI, with a potential impact on treatment efficacy [117, 118]. Hence, weight loss should be encouraged in obese and overweight breast cancer survivors. Moreover, the weight loss response to anti-obesity medications (i.e. liraglutide, semaglutide and phentermine) seems poorer in obese breast cancer patients treated with aromatase inhibitors when compared to obese patients without breast tumours [119]. A combination of regular physical exercise, diet, and cognitive behavioural has demonstrated to be highly effective. Harvie et al. randomized 243 overweight patients and 166 normal weight patients in a three arms trial (a 3-month home unsupervised programme, a supervised community programme, and a control arm receiving standard written advice). Patients assigned to both interventional arms experienced a reduction of weight and body fat and an increase in terms of physical activity levels; a reduction in cardiovascular disease markers was found only in the supervised group [120]. Among the interventions, eHealth tools seem to be particularly appealing due to their wide availability and low cost. However, they are still under investigation.

## Conclusions

OFS is standard strategy for ovarian function preservation in premenopausal women receiving chemotherapy and is a key component of the adjuvant endocrine treatment for most patients with hormone receptor-positive breast cancer, particularly in those at intermediate and high risk of relapse. Nevertheless, the side effects of OFS may be highly impactful and long-lasting. A correct counselling and proactive management of the side effects of OFS should be considered a relevant part of survivorship care to improve patients’ quality of life and treatment adherence.

## Data Availability

Not applicable.
